# Variability of Organophosphorous Pesticide Metabolite Levels in Spot and 24-hr Urine Samples Collected from Young Children during 1 Week

**DOI:** 10.1289/ehp.1104808

**Published:** 2012-10-09

**Authors:** Asa Bradman, Katherine Kogut, Ellen A. Eisen, Nicholas P. Jewell, Lesliam Quirós-Alcalá, Rosemary Castorina, Jonathan Chevrier, Nina T. Holland, Dana Boyd Barr, Geri Kavanagh-Baird, Brenda Eskenazi

**Affiliations:** 1 Center for Environmental Research and Children’s Health; 2 Division of Environmental Health Sciences, and; 3 Division of Biostatistics, School of Public Health, University of California, Berkeley, Berkeley, California, USA; 4 Department of Environmental Health, Rollins School of Public Health, Emory University, Atlanta, Georgia, USA

**Keywords:** biomarkers, children, exposure, metabolites, organophosphorous, pesticides, urine, variability

## Abstract

Background: Dialkyl phosphate (DAP) metabolites in spot urine samples are frequently used to characterize children’s exposures to organophosphorous (OP) pesticides. However, variable exposure and short biological half-lives of OP pesticides could result in highly variable measurements, leading to exposure misclassification.

Objective: We examined within- and between-child variability in DAP metabolites in urine samples collected during 1 week.

Methods: We collected spot urine samples over 7 consecutive days from 25 children (3–6 years of age). On two of the days, we collected 24-hr voids. We assessed the reproducibility of urinary DAP metabolite concentrations and evaluated the sensitivity and specificity of spot urine samples as predictors of high (top 20%) or elevated (top 40%) weekly average DAP metabolite concentrations.

Results: Within-child variance exceeded between-child variance by a factor of two to eight, depending on metabolite grouping. Although total DAP concentrations in single spot urine samples were moderately to strongly associated with concentrations in same-day 24-hr samples (*r* ≈ 0.6–0.8, *p* < 0.01), concentrations in spot samples collected > 1 day apart and in 24-hr samples collected 3 days apart were weakly correlated (*r* ≈ –0.21 to 0.38). Single spot samples predicted high (top 20%) and elevated (top 40%) full-week average total DAP excretion with only moderate sensitivity (≈ 0.52 and ≈ 0.67, respectively) but relatively high specificity (≈ 0.88 and ≈ 0.78, respectively).

Conclusions: The high variability we observed in children’s DAP metabolite concentrations suggests that single-day urine samples provide only a brief snapshot of exposure. Sensitivity analyses suggest that classification of cumulative OP exposure based on spot samples is prone to type 2 classification errors.

Spot urine samples have been used to assess organophosphorous (OP) pesticide exposures in epidemiological and biomonitoring studies, with samples analyzed for either nonspecific dialkyl phosphate (DAP) metabolites [Centers for Disease Control and Prevention (CDC) 2003] or pesticide-specific metabolites (Fenske et al. 2002). Because collection of spot urine samples is relatively simple and noninvasive, this sample type is commonly used in studies involving children (Fenske et al. 2005). Several studies suggest that spot samples provide meaningful measures of exposure, reporting associations, for example, between residence near farmlands and OP metabolite levels in children’s urine (Fenske et al. 2000). However, concerns have been raised as to whether metabolites measured in a single spot sample accurately reflect exposure for the full day or beyond.

Several factors may result in exposure misclassification when using spot samples: *a*) Because the concentration of a chemical, urine volume, and rate of urinary excretion vary with fluid and salt intake, time of day, and other factors (Barr et al. 2005; Boeniger et al. 1993; Cornelis et al. 1996), exposure measurements may be more variable than other sample types, such as first morning voids (FMVs) (Kissel et al. 2005), which are more concentrated and reflect a longer period of accumulation, or 24-hr urine collections, considered by some as the “gold standard” (Aprea et al. 2002; Boeniger et al. 1993; Fenske and Elkner 1990; Martin et al. 1996); *b*) because of within-child variability in creatinine excretion (Barr et al. 2005; Boeniger et al. 1993), creatinine adjustment may introduce new variability that does not reflect urine dilution; and *c*) most OP pesticides have biological half-lives of only 12–36 hr (Needham 2005) and are metabolized and excreted from the body in hours to days (Barr 2008). Thus, OP pesticide metabolite excretion in a brief sampling period, whether measured in a spot, FMV, or 24-hr urine sample, may not accurately represent a child’s cumulative OP pesticide exposure over longer periods.

One recent study of 44 preschool-age children who provided between 10 and 26 biweekly spot urine samples over 21 months reported that within-child variability for the five DAP metabolites measured exceeded between-child variability by several factors (Griffith et al. 2011)—a finding that underscores concern about the reliability of this measure and raises questions about the source of within-child variability. To date, no studies have evaluated variability of OP metabolites in within-day samples or in 24-hr urine samples collected several days apart. Nor have studies compared variability in DAP measurements when expressed in terms of unadjusted concentrations, creatinine-adjusted concentrations, or urinary excretion rates.

To address these questions, in this study with preschool-aged children in California we aimed to *a*) evaluate the reproducibility of children’s nonspecific OP pesticide measurements within a 1-week period; *b*) evaluate the influence of concentration corrections (i.e., creatinine adjustment and use of urinary excretion rate) on reproducibility of DAP measurements; *c*) determine the degree of correspondence between pesticide metabolite concentration in spot urine samples (including FMVs) and same-day 24-hr samples; and *d*) evaluate the sensitivity and specificity of spot urine samples to classify full-week average measurements.

## Methods

*Study population.* We recruited a convenience sample of 25 children (10 boys, 15 girls) from clinics serving low-income families in the Salinas Valley, an agricultural region. Eligible children were between 3 and 6 years old, in good health with no history of diabetes or renal disease, toilet-trained, free of enuresis, and had English- or Spanish-speaking mothers who were at least 18 years old. Sampling occurred in March and April 2004. The study was approved by the University of California at Berkeley Committee for the Protection of Human Subjects, and parents provided written informed consent.

*Urine sample collection.* Each family participated for 7 consecutive days (study days 1–7). On day 1, study staff provided families with sampling supplies and instructions on sample collection. Supplies included specimen trays (cleaned, sterile Specipan™; Baxter Scientific, McGaw Park, IL), gloves, collection jars with blank labels, a small refrigerator, and two 24-hr sampling record forms. On spot-sampling days (1, 3, 4, 6, and 7), families collected a single void at their convenience; families sometimes collected an FMV as the spot sample. On 24-hr sampling days (2 and 5), families were instructed to collect the child’s FMV, all daytime and evening spot voids, and the FMV of the following day as separate specimens, and to note the timing of all voids (including missed voids) during this period on the 24-hr sampling record form.

Sample collection methods have been previously validated in this age range (Lu et al. 2001). Children voided directly into a collection jar or into a clean, sterile specimen tray placed on a training potty or toilet. If samples were collected in specimen trays, parents transferred specimens into collection jars. Parents identified each sample as an FMV or a non-FMV spot sample, recorded the time of collection on jar labels, and stored specimens in the refrigerator until daily collection by research staff. On 24-hr sampling days, research staff reviewed the sampling record form with the families to assure its accuracy and completeness.

Twenty-four-hour samples included 3–11 collected voids (mean, 6.5). Overall, 86% (range, 50–100%) of the reported voids during the 24-hr sampling periods were collected. Twenty-two (44%) of the 24-hr samples were based on 100% collection of all voids. Reasons for missed voids included out-of-home bathroom use, toileting accidents, and participant error, such as missing an evening void. In sensitivity analyses, we excluded seven 24-hr samples with fewer than five total voids to confirm that collection errors did not substantially change study results (not shown).

*Sample processing and analysis.* Samples were processed at the Salinas Valley study office. The weight of each void was measured (grams), and the volume (milliliters) was estimated assuming a urine specific gravity of 1.022 (Salita et al. 1998). Individual voids from 24-hr sampling sessions that were not selected for individual analysis were pooled. After aliquotting, samples were stored at –80°C until being shipped on dry ice to the CDC for analysis.

We measured six DAP metabolites in all samples: three dimethyl (DM) phosphate metabolites—dimethylphosphate (DMP), dimethylthiophosphate (DMTP), and dimethyldithiophosphate (DMDTP); and three diethyl (DE) phosphates—diethylphosphate (DEP), diethylthiophosphate (DETP), and diethyldithiophosphate (DEDTP). These metabolites represent approximately 81% of the agricultural OP pesticides used in the Salinas Valley (Bradman et al. 2007). Laboratory methods and quality control procedures are described in detail elsewhere (Bravo et al. 2004). Briefly, samples were lyophilized to remove water, redissolved in a 1:1 solution of acetonitrile and diethyl ether, and analyzed using gas chromatography–tandem mass spectrometry using isotope dilution. Creatinine concentrations were determined using a commercially available method (Vitros CREA slides; Ortho Clinical Diagnostics, Raritan, NJ). Westgard rules for quality control were used to establish the validity of each analytical run (Westgard 2003). Limits of detection (LODs) were 0.2 μg/L for all DEs, 0.5 μg/L for DMP, 0.4 μg/L for DMTP, and 0.1 μg/L for DMDTP.

Levels below the LOD were imputed as LOD/_√_^–^2 (Hornung and Reed 1990), and molar concentrations were summed within each sample to yield total DM, total DE, and total DAP concentrations. Metabolite levels in 24-hr voids were computed as the volume-weighted average of concentrations in all samples collected on sampling days 2 and 5, which included the FMV samples from the following day. We expressed metabolite concentration and/or excretion in three ways: *a*) unadjusted metabolite concentration (nanomoles metabolite per liter of urine); *b*) creatinine-adjusted metabolite concentration (nanomoles metabolite per gram of creatinine); and *c*) urinary excretion rate (UER) of each metabolite (nanomoles metabolite per minute) computed as the total moles of excreted metabolite divided by the duration of time in minutes since the previous void. For 24-hr collections, time since the previous void was calculated as the time elapsed between the last void preceding the 24-hr sample collection (typically the last void on day 1 or 4) and the last void included in the 24-hr sample (typically the FMV on day 3 or 6).

## Data Analysis

Statistical analyses were performed using Stata 10 for Windows (StataCorp LP, College Station, TX). We first computed descriptive statistics and assessed normality. Log_10_-transformed values were used for subsequent analyses.

*Correspondence between spot samples and 24-hr samples.* We constructed generalized estimating equation (GEE) models using DAP concentration in a 24-hr sample as the outcome variable and concentration in a same-day spot sample (FMV or non-FMV) or a combination of these two spot samples as the predictor variable. Combinations of two spot samples were modeled as separate predictor variables and as a single variable computed as either the arithmetic or volume-weighted average of the individual samples. Analyses using each DAP variable type (i.e., unadjusted concentrations, creatinine-adjusted concentrations, and UER) were compared. Robust standard errors were calculated.

*Reproducibility of spot and 24-hr urine samples.* We used mixed random-effects models to compute the between-child and within-child variance for 24-hr samples from days 2 and 5, and for all FMV spot samples. For all non-FMV spot samples and for all spot samples (FMV and non-FMV), we estimated the variance attributable to each of three nested components—between-child, within-child, and within-child within-day variability (because multiple samples were available from the same day)—using two-level random-intercept models (Marchenko 2006). This method parses the total within-child variance into two parts: the component attributable to same-day within-child variability, and the component attributable to between-day within-child variability (Hauser et al. 2004; Preau et al. 2010; Ye et al. 2011). We calculated Akaike Information Criterion (AIC) values for the nested models with the largest number of observations (the all spot samples model) to compare the fit of models using unadjusted DAP concentrations, creatinine-adjusted concentrations, and UER. We also calculated the intraclass correlation coefficient (ICC) for each group of samples. The ICC is the ratio of between-subject variance to total variance.

*Correlation of spot samples collected 0–6 days apart.* We computed Pearson’s correlations between pairs of samples collected on the same day (0 days apart) as well as on pairs of samples collected between 1 and 6 days apart. This analysis included all possible unique pairings of each child’s spot samples, and was conducted separately for pairs of FMV spots only, pairs of non-FMV spots only, and for all pairs of spots regardless of FMV status. Tests of significance were computed using a robust estimate of the variance.

*Sensitivity and specificity.* We evaluated the sensitivity and specificity of one, two, or three randomly-selected spot urine samples from each child as predictors of high (top 20%) or elevated (top 40%) weekly average DAP metabolite concentrations. For “true” exposure, we calculated the arithmetic mean metabolite concentration of all spot urine samples collected from each child during the week, categorized these in quintiles, and assigned “true” high (top 20%) or elevated (top 40%) exposure level. For predictor sets, we created 10 data sets each containing one randomly selected spot sample per child (i.e., 25 observations total per data set). Within each randomly-selected data set, we again categorized the 25 metabolite concentration in quintiles, and assigned “predicted” high (top 20%) or elevated (top 40%) exposure levels. This process mimicked the exposure classification process in epidemiological studies, in which knowledge of the range and rankings of exposure in a population is often limited to what can be observed in the single sample or small number of samples collected per subject. The sensitivity and specificity figures we report represent the average sensitivity and specificity observed across the 10 separate random samples. We report findings separately for FMV samples only, for non-FMV samples only, and for all spot samples. To determine whether collection of more than one spot sample could improve sensitivity, we repeated this analysis using the arithmetic mean of two or three spot samples from each child collected on different days. Because some children lacked three FMV or non-FMV samples from different days, we only assessed the sensitivity and specificity of three spot samples in the “any spot sample” group.

## Results

All children were Mexican American, and their ages ranged from 3 to 6.5 years (mean ± SD age, 4.5 ± 0.93 years). Children urinated between 3 and 12 times (mean, 5.7 voids) per 24-hr period, and the volume of their individual spot samples ranged from 4.8 to 642.2 mL (mean, 146.6 mL) for FMV samples and from 8.4 to 238.3 mL (mean, 68.1 mL) for non-FMV spot samples. The creatinine concentration of spot samples ranged from 9.4 to 213.2 mg/dL (mean, 86.6 mg/dL) for FMV samples and from 4.5 to 158.7 mg/dL (mean, 68.6 mg/dL) for non-FMV samples. Creatinine concentration varied more within children than between children for both FMV (ICC = 0.37) and non-FMV (ICC = 0.22) spot samples. Individual DE and DM metabolites were detected in > 70% of samples, except for DEDTP (17%) (data not shown). Total DM concentrations exceeded total DE concentrations, and total DAP levels reflected predominantly DM metabolites (Table 1). Central tendency measures (e.g., means, medians) for unadjusted concentration values were similar for FMV spot samples and 24-hr samples, which were generally higher than levels in non-FMV spot samples. In contrast, central tendency measures for creatinine-adjusted concentration values were more similar for FMVs and non-FMV spot samples, with both generally lower than creatinine-adjusted 24-hr samples.

**Table 1:** Unadjusted and creatinine-adjusted DAP concentrations in urine samples.

Type of sample	DF (%)	Unadjusted							Creatinine adjusted						
		GM	Mean	Median	Max	GM	Mean	Median	Max
Non-FMV spot samples (n = 137)																		
Total DAPs	99.3	110	239	122	4,820	196	529	218	20,600
Total DMs	94.2	63.0	179	60.3	4,790	112	436	96.2	20,500
Total DEs	95.6	26.8	59.8	31.1	500	47.7	98.8	68.0	529
FMV samples (n = 110)																		
Total DAPs	98.2	162	307	157	2,530	205	483	200	11,800
Total DMs	95.5	92.3	234	94.4	2,380	117	392	124	11,600
Total DEs	97.3	43.4	72.5	57.1	391	55.1	91.1	77.6	376
24-hr samples (n = 50)																		
Total DAPs	—	158.0	296	144	3,700	275	621	245	10,100
Total DMs	—	94.6	230	89.9	3,590	166	508	139	9,920
Total DEs	—	45.9	65.0	53.3	248	78.9	113	93.6	610
Abbreviations: DF, detection frequency; GM, geometric mean; Max, maximum.																		

**Figure 1:**
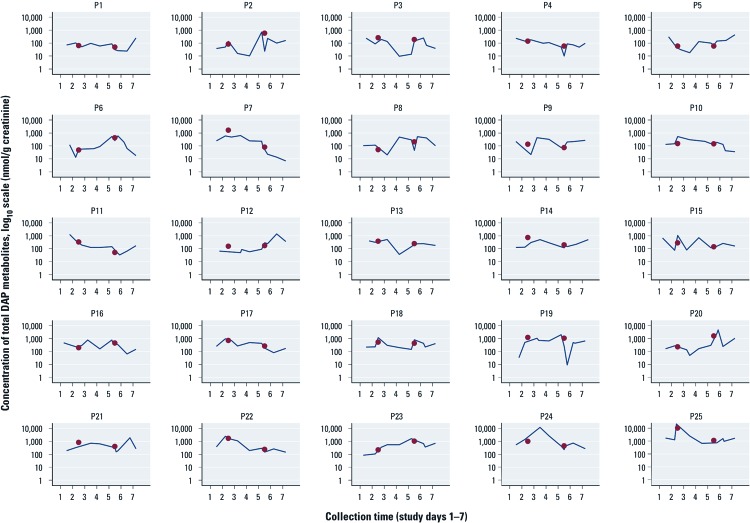
Concentration of total DAP metabolites (nmol/ gram creatinine) in log_10_ scale for all spot samples and 24-hr samples collected over 1 week. Each panel represents an individual participant (*n* = 25; P1–P25.) The dots in each panel represent the total DAP metabolite concentration in the 24-hr samples from sampling days 2 and 5, respectively. The panels are ranked in order of ascending arithmetic mean of all spot samples collected for each child. Thus, the bottom row (P21–P25) contains the children rated as having “true” high (top 20%) weekly exposure in the sensitivity and specificity analysis, and the bottom two rows exposure.

Figure 1 presents the total DAP metabolite concentrations [in log_10_ scale (nanamoles per gram creatinine)] for each participant for all spot samples collected over the 7 consecutive sampling days. There are no clear exposure trends over time among the children, though our data indicate that shifts of up to two orders of magnitude can occur over the week (Figure 1, P7) or within a single day (Figure 1, P19, day 5). Twenty-four-hour samples collected only 3 days apart can likewise differ by an order of magnitude (e.g., Figure 1, P2, P7, P25).

Table 2 presents results of GEE models examining how well same-day spot samples predict 24-hr metabolite levels. For models examining a single unadjusted spot sample (FMV or non-FMV) and its respective 24-hr sample, the predictive power of the model, defined by the coefficient of determination (*R*^2^), was highest for FMVs [*R*^2^ for total DAP metabolites (nmol/L) = 0.54 for FMVs vs. 0.39 for non-FMV spots]. The predictive power of the model was higher for creatinine-adjusted metabolites compared with unadjusted metabolites, especially for non-FMVs, with little difference between the sample types (total DAP *R*^2^ = 0.57 for FMV and 0.63 for non-FMVs). The predictive power was lowest for UER models.

**Table 2:** Modeling of 24-hr metabolite excretion^*a*^ using same-day spot urine samples as predictors (*n* = 41^b^).

Type of spot sample/metabolite excretion unitsc		Metabolite type	Regression model						Pearson correlation		
			β (95% CI)		Intercept	Model R2	r	p-Value
Non-FMV spot	
Unadjusted (nmol/L)		Total DAPs	0.52	(0.28, 0.76)	1.11	0.39	0.63	< 0.001
		Total DMs	0.60	(0.41, 0.78)	0.86	0.49	0.70	< 0.001
		Total DEs	0.25	(0.06, 0.43)	1.30	0.14	0.37	0.011
Creatinine adjusted (nmol/g creatinine)		Total DAPs	0.66	(0.48, 0.84)	0.87	0.63	0.80	< 0.001
		Total DMs	0.69	(0.53, 0.84)	0.74	0.68	0.82	< 0.001
		Total DEs	0.33	(0.16, 0.50)	1.31	0.25	0.50	< 0.001
Urinary excretion rate (nmol/min)		Total DAPs	0.51	(0.23, 0.79)	–0.69	0.42	0.65	0.001
		Total DMs	0.57	(0.37, 0.77)	–0.72	0.52	0.72	< 0.001
		Total DEs	0.21	(0.01, 0.40)	–1.49	0.09	0.30	0.036
FMV spot			
Unadjusted (nmol/L)		Total DAPs	0.66	(0.42, 0.90)	0.70	0.54	0.74	< 0.001
		Total DMs	0.66	(0.42, 0.89)	0.66	0.57	0.75	< 0.001
		Total DEs	0.39	(0.18, 0.60)	0.99	0.26	0.51	0.001
Creatinine-adjusted (nmol/g creatinine)		Total DAPs	0.75	(0.50, 1.01)	0.68	0.57	0.76	< 0.001
		Total DMs	0.72	(0.47, 0.97)	0.71	0.59	0.77	< 0.001
		Total DEs	0.41	(0.19, 0.63)	1.15	0.27	0.52	0.001
Urinary excretion rate (nmol/min)		Total DAPs	0.43	(0.13, 0.74)	–0.72	0.37	0.60	0.008
		Total DMs	0.51	(0.22, 0.79)	–0.71	0.45	0.67	0.001
		Total DEs	0.26	(0.04, 0.49)	–1.36	0.19	0.43	0.025
Average of non-FMV and FMV spotsd			
Unadjusted (nmol/L)		Total DAPs	0.90	(0.76, 1.05)	0.17	0.79	0.89	< 0.001
		Total DMs	0.92	(0.78, 1.07)	0.09	0.84	0.92	< 0.001
		Total DEs	0.66	(0.78, 1.07)	0.57	0.44	0.66	< 0.001
Creatinine-adjusted (nmol/g creatinine)		Total DAPs	0.92	(0.81, 1.03)	0.20	0.84	0.92	< 0.001
		Total DMs	0.93	(0.81, 1.04)	0.14	0.89	0.94	< 0.001
		Total DEs	0.70	(0.44, 0.96)	0.60	0.48	0.69	< 0.001
Urinary excretion rate (nmol/min)		Total DAPs	0.83	(0.64, 1.03)	–0.30	0.77	0.88	< 0.001
		Total DMs	0.85	(0.69, 1.01)	–0.32	0.82	0.91	< 0.001
		Total DEs	0.66	(0.34, 0.99)	–0.67	0.39	0.62	< 0.001
aSpot and 24-hr samples analyzed on log10 scale. bn = 41 child-days; 9 samples lacked an FMV sample and were excluded. Regression models are clustered by child. cUnits of both the spot sample(s) and the 24-hr sample. dValues converted to log10 scale after arithmetic average of concentrations on normal scale was computed; not volume-weighted.														

The best-fitting models were obtained when the arithmetic mean of an FMV and a non-FMV sample was used to predict the 24-hr values (Table 2). The best model fit was observed for creatinine-adjusted values (total DAP *R*^2^ = 0.84), but the variability in 24-hr metabolite levels explained by unadjusted values and UER estimates were comparable (*R*^2^ = 0.79 and 0.77, respectively). Model fit was strongest for analyses of total DM metabolites, and weakest for total DE metabolites. Volume-weighted averages of FMV and non-FMV spots produced model fits that were similar to that obtained with the arithmetic mean (data not shown). Results of sensitivity analyses that excluded seven 24-hr samples with fewer than five spot samples were consistent with those described above (data not shown).

AIC values for mixed random-effects models used to estimate the variance in total DAPs attributable to between-child versus within-child sources based on all spot urine samples (*n* = 247) indicated that best model fit was achieved with creatinine-adjusted values (AIC = 364; Table 3) versus AIC = 372 and 451, respectively, for unadjusted concentration and UER models [see Supplemental Material, Table S1 (http://dx.doi.org/10.1289/ehp.1104808)]. Relative to unadjusted concentration and UER models, use of creatinine-adjusted values also provided the greatest degree of distinction between individual children [e.g., ICC = 0.32 for creatinine-adjusted total DAP metabolites in the “any spot sample” model (Table 3) vs. ICC = 0.21 and 0.15, respectively, for the analogous unadjusted concentration and UER models (see Supplemental Material, Table S1)].

**Table 3:** Variance apportionment of log-transformed creatinine-adjusted DAP metabolite concentrations in spot urine samples collected during 1 week and in 24-hr voids collected 3 days apart (*n* = 25 children).

Type of sample	na	Total DAPs					Total DMs					Total DEs				
		Variance	Percent total variance	ICC	Variance	Percent total variance	ICC	Variance	Percent total variance	ICC
Non-FMV spot samples	137
Between child	0.075	25	0.27b	0.114	27	0.30b	0.041	12	0.14b
Within child, between day	0.105	34	0.149	35	0.108	31
Within child, within day	0.125	41	0.162	38	0.198	57
FMV samples	110
Between child	0.105	34	0.34	0.172	38	0.39	0.035	12	0.11
Within childc	0.207	66	0.276	62	0.246	88
Any spot samples (FMV or Non-FMV)	247
Between child	0.097	31	0.32b	0.157	36	0.37b	0.033	10	0.11b
Within child, between day	0.064	20	0.070	16	0.049	15
Within child, within day	0.152	49	0.213	48	0.238	74
24-hr voids	50
Between child	0.089	35	0.35	0.131	36	0.026	16	0.16
Within childc	0.168	65	0.237	64	0.132	84
aNo. of samples used in calculation. bRatio of between-child to total variability as calculated using a one-factor (child) as opposed to a two-factor nested mixed-effects model. cBecause FMV spots and 24-hr voids allow only one measure per day, the distinction between within-day versus between-day variability is not applicable.																				

Among creatinine-adjusted total DAP concentration models (Table 3), non-FMV spot samples displayed the poorest reproducibility (ICC = 0.27 for creatinine-adjusted total DAP metabolites). Inclusion of FMV samples in this analysis (the “any spot sample” set) modestly increased the between-child relative to within-child variance (ICC = 0.32) and suggested somewhat more between-day stability in measures (i.e., smaller within-child between-day variance). However, examination of 24-hr voids collected 3 days apart indicates that this between-day stability was limited, with 65% of total variance attributed to within-child variability (Table 3). A sensitivity analysis limited to the most complete 24-hr samples (based on five or more spot samples) indicated even greater within-child variance (for total DAPs, 77% of total, ICC = 0.23; data not shown). The variance components of creatinine-adjusted total DM metabolites (Table 3) were similar to those for total DAP metabolites, though the reproducibility of DM samples was slightly higher (ICCs = 0.30–0.39, compared with 0.27–0.35 for total DAPs). Reproducibility of creatinine-adjusted DE metabolites was much lower (ICCs = 0.11–0.16) (Table 3), and within-child variance exceeded between-child variance between a factor between five (e.g., 57% vs. 12% of the total variance for non-FMV spot samples) and eight (e.g., 88% vs. 12% of the total variance for FMV spot samples) for these metabolites.

On the basis of their superior model performance in the preceding analyses, we used creatinine-adjusted values in subsequent analyses.

Table 4 presents Pearson correlations of creatinine-adjusted metabolite levels in spot samples collected 0–6 days apart. Correlations for total DAPs and total DMs were moderate (≈ 0.5) and statistically significant for samples collected on the same day (i.e., 0 days apart) or 1 day apart, and became weaker as the number of days between samples increased. All correlations were weak and not significant for samples collected 5–6 days apart. DE metabolites had weaker correlations compared with DMs, and the decay in correlations across days was more rapid for DEs than for DM or total DAP metabolites. FMV samples tended to have higher correlations than non-FMV samples or any spot samples. The correlations between samples collected 2–3 days apart are comparable with Pearson correlations between levels in 24-hr samples collected 3 days apart, which were 0.35. 0.36, and 0.15, respectively, for total DAP, DM, and DE metabolites.

**Table 4:** Pearson correlations of creatinine-adjusted DAP metabolite concentrations (log_10_ scale) in paired, same-child spot urine samples collected 0–6 days apart.

Type of spot sample metabolitetype	Days elapsed between paired samples												
	0 (same day)	1	2	3	4	5	6
Non-FMV spot samples	n = 26a	84	60	58	65	24	2
Total DAPs	0.54*	0.48*	0.08	0.24	0.16	–0.01	—
Total DMs	0.58*	0.45*	0.27*	0.37*	0.10	–0.15	—
Total DEs	0.44*	0.34*	–0.21	0.13	0.13	0.31	—
FMV samples	n = 0a	58	57	45	20	21	2
Total DAPs	—	0.54*	0.27*	0.27	0.26	0.25	—
Total DMs	—	0.59*	0.34*	0.30*	0.38	0.31	—
Total DEs	—	0.25	–0.01	0.32*	–0.10	–0.05	—
Any spot samples (FMV or Non-FMV)	n = 92a	303	248	203	154	77	25
Total DAPs	0.46*	0.45*	0.25*	0.23*	0.17*	0.16	0.16
Total DMs	0.49*	0.48*	0.35*	0.30*	0.21*	0.18	0.13
Total DEs	0.25*	0.22*	–0.15	0.08	0.02	0.11	–0.27
—, not reported due to small size. aUnique, within-child pairings of spot samples that meet pairing requirements (e.g., n = 92 discrete pairings of within-child spots collected on the same day, or 0 days apart). *p ≤ 0.05.														

Table 5 presents results of sensitivity and specificity analyses. In this sample of participants, children with high (top 20%) weekly average total DAP metabolite concentrations would be correctly classified by a single non-FMV spot sample 52% of the time. Using two samples increased the sensitivity marginally to 56%. FMVs also showed marginally higher sensitivity than non-FMV samples. Within the “any spot sample group,” three-spot predictors offered little apparent advantage over two-spot predictors. Overall, the sensitivity was higher to classify children in the top 40th percentile (elevated) than in the top 20th percentile, with a slight improvement with two versus one sample. Specificity was uniformly higher than sensitivity. Use of a single non-FMV spot sample would correctly identify a child in the lower 80th percentile (i.e., not “high”) 88% of the time, and would identify a child in the lower 60th percentile (i.e. not “elevated”) 78% of the time.

**Table 5:** Sensitivity and specificity^*a*^ for classifying children with high (top 20%) and elevated (top 40%) one-week average total DAP metabolite concentrations^*b*^ with one, two, or three urine samples.

Type of sample	High (top 20%)			Elevated (top 40%)		
	Sensitivity	Specificity	Sensitivity	Specificity
Non-FMV spot samples
One sample	0.52	0.88	0.67	0.78
Two samplesc	0.56	0.89	0.68	0.79
FMV samples
One sample	0.58	0.90	0.65	0.77
Two samplesc	0.58	0.90	0.78	0.85
Any spot samples (FMV or Non-FMV)
One sample	0.46	0.87	0.63	0.75
Two samplesc	0.60	0.90	0.76	0.84
Three samplesc	0.64	0.91	0.73	0.82
aAverage sensitivity and specificity calculated based on 10 separate random samples of predictor spot samples. bCalculations use creatinine-adjusted total DAP metabolite concentration (nmol/g creatinine) on the log10 scale. cPairs and trios consist of spot samples collected on different days (i.e., no pairs of same-day spots).								

## Discussion

This study of variability in DAP metabolites of OP pesticides in urine samples from children 3–6 years old indicates high variability over a 1-week time frame. Although we observed strong correlations between full-day 24-hr samples and same-day spot samples (see Pearson correlations, Table 2), there was weak correlation between DAP metabolite levels in 24-hr samples collected 3 days apart or in spot samples collected > 1 day apart, with correlations weaker for DE compared with DM DAP metabolites. Further, within-child variance was approximately two to three times the between-child variance for total DAP metabolites (e.g., 66% vs. 34% for FMV samples for creatinine-adjusted total DAPs, ICC = 0.27–0.35). This ratio was lower (and thus, the ICC higher) in DM metabolites compared with DE metabolites (ICC = 0.30–0.39 vs. ICC = 0.11–0.16, respectively). Finally, we found that DAPs measured in one, two, or even three spot urine samples have relatively low sensitivity to identify children who would be considered the most highly exposed on the basis of their average full-week DAP concentrations. For example, a single non-FMV spot sample would correctly identify only 52% of the children whose true weekly exposure was in the top quintile, suggesting high type 2 classification errors; by contrast, spot samples appear to offer good specificity, with a single non-FMV spot sample correctly identifying about 88% of children with true total DAP exposure below the top quintile. Overall, the high within-child variance, the weak correlation across days, and low specificity suggests that single-day measurements may not adequately characterize exposure for longer time frames necessary for chronic risk assessments or epidemiologic studies.

Our analysis replicated methods applied by Ye et al. (2011) and Preau et al. (2010) to partition the variance components among between-child, within-child between-day, and within-child within-day components for sample types with more than one sample per day (Table 3). Similar to their findings for bisphenol A and monoethyl phthalate, we found that overall within-person variance was higher than between-person variance, and that within-day variance was the largest component of total variance, suggesting that differences in exposure between days were lower compared with fluctuations that occurred during the course of each day. This finding suggests that eliminating within-day variability would substantially reduce within-child relative to between-child variability overall. However, we found that 65% of the total variance in 24-hr urine samples collected 3 days apart was due to within-child between-day variability, even though the 24-hr samples reflect complete sampling with no within-day variability (i.e., the entire sample was collected). These apparently contradictory findings suggest to us that the variance component analyses may overestimate the within-person within-day variance component. Specifically, we suspect the models may overestimate the degree of within-day variability that is biologically plausible, given that the total number of possible within-day observations is capped by the number of times individuals urinate each day, which in this study ranged between 3 and 12 times.

Our study provides important information about the appropriateness of creatinine adjustment to control for urinary dilution in children 3–6 years of age. Overall, our findings suggest that creatinine adjustment of urinary DAP concentrations in children ages 3–6 years maximizes between-child relative to within-child variance (thus reducing exposure misclassification), improves estimation of the average 24-hr DAP concentration on the basis of spot urine samples, and may decrease the difference between FMV and easier-to-collect non-FMV spot samples in terms of their ability to estimate total 24-hr excretion. Despite this empirical evidence suggesting that creatinine adjustment effectively normalized the metabolite concentrations, this method may introduce other sources of variability. Specifically, creatinine varies as a function of sex, age, meat consumption, body size, and muscle mass (Boeniger et al. 1993). Thus, large differences in size, muscle mass, and diet between developing children of the same age could result in very different normalized metabolite values among children with the same exposure. In future analyses, we will examine creatinine excretion patterns in these children and assess adjustment for specific gravity (not yet quantified in these samples) as an alternative approach to account for urinary dilution.

In summary, our findings raise concerns about the utility of urinary DAP metabolites as a biomarker of chronic OP pesticide exposure in young children. Two recent published analyses from our Center have detected no association between DAP measures in children and adverse outcomes, although they did detect significant associations between maternal prenatal DAP measurements and child neurodevelopmental outcomes (Bouchard et al. 2011; Marks et al. 2010), despite the fact the we have also observed high within versus between variability in maternal urine (Bradman et al. 2005). One interpretation of this finding is that OP exposures in childhood are less critical than exposures that occur *in utero*: The fetus is so exceptionally sensitive to OPs that the strength of the prenatal exposure effects are evident despite limitations in the exposure measure. The current study suggests another plausible explanation: that misclassification of child OP exposure biased results toward the null hypothesis.

Our study has several limitations. Collecting 24-hr urine samples from young children proved challenging, and missed voids due to occasional toileting accidents or other circumstances were beyond the control of study staff. Though 86% of the voids reported to have occurred during 24-hr sampling periods were collected, missed voids meant that individual spot samples made up a greater proportion of the 24-hr volume-weighted average than they should have. Thus, our estimates of the association between metabolites in spot and 24-hr samples (Table 2) represent an upper bound. We measured class-specific organophosphate pesticide DAP metabolites, and our findings should not be generalized to pesticide-specific OP metabolites, which may exhibit different variance patterns. Our study was also conducted in an agricultural community during the growing season, and it is possible that intermittent exposures related to nearby agricultural pesticide use may have caused higher variability in metabolite levels than would be observed in the general U.S. population. Furthermore, different exposure scenarios could result in different variability patterns. Before the phaseout of diazinon and chlorpyrifos in household pesticides (U.S. Environmental Protection Agency 2000, 2001), for example, children from homes with frequent indoor use of such pesticides may have shown more stable exposure levels. In a follow-up analysis, we intend to use additional data available to us to assess which potential exposure sources (e.g., diet, nearby agricultural pesticide use) best account for the metabolite variability patterns we present here.

## Conclusions

We found that though DAP metabolites in single or multiple spot samples are strongly correlated with levels in same-day 24-hr samples, children’s urinary DAP concentrations vary greatly from day to day, and use of spot samples to characterize a child’s cumulative weekly exposure results in a moderate degree of misclassification. Exposure misclassification resulting from urinary metabolite variability has the potential to bias measures of association between early childhood OP exposures and developmental outcomes in epidemiologic research toward the null hypothesis; such misclassification may account for the weak or null findings often reported to date between pediatric urinary DAP concentrations and child development (Bouchard et al. 2011; Marks et al. 2010). Additional research on variability in measures of nonpersistent compounds in children is needed to assure that exposure biomarkers are valid and that epidemiological studies have adequate power to detect health outcomes that may result from these exposures.

## Supplemental Material

(12 KB) PDF
