# Supplementary material for: Variability of Organophosphorous Pesticide Metabolite Levels in Spot and 24-hr Urine Samples Collected from Young Children during 1 Week
Source: Environ Health Perspect. 2012 Oct 9;121(1):118–24. doi: 10.1289/ehp.1104808 (PMC3553429; doi:10.1289/ehp.1104808)
Supplement: (12 KB) PDF [file ehp.1104808.s001.pdf]

Supplemental Material

Variability of Organophosphorous Pesticide Metabolite Levels in Spot and 24-hr Urine Samples Collected from Young Children during 1 Week

Asa Bradman*[1], Katherine Kogut*[1], Ellen A. Eisen[1,2] , Nicholas P. Jewell[1,3], Lesliam Quirós-Alcalá[1], Rosemary Castorina[1], Jonathan Chevrier[1], Nina T. Holland[1], Dana Boyd Barr[4], Geri Kavanagh-Baird[1], Brenda Eskenazi[1]

*A. Bradman and K. Kogut share lead authorship.

Supplemental Table S1. The variance apportionment of log-transformed unadjusted total DAP metabolite concentrations and log-transformed urinary excretion rate (UER) values in spot urine samples collected during one week and in 24-hour voids collected 3 days apart (N=25 children)

		Unadjusted Total DAPs			Total DAPs UER		
**Type of Sample**	**N [a]**	**Variance**	**% Total Variance**	**ICC**	**Variance**	**% Total Variance**	**ICC**
**Non-FMV Spot Samples**							
Between child	137	0.043	16%	0.16[b]	0.036	10%	0.10[b]
Within child, between day		0.073	26%		0.155	43%	
Within child, within day		0.162	58%		0.171	47%	
**First Morning Void (FMV) Samples**							
Between child	110	0.082	30%	0.30	0.046	12%	0.13
Within child[c]		0.194	70%		0.334%	88%	
**Any Spot Samples (FMV or Non-FMV)**							
Between child	247	0.061	21%	0.21[b]	0.056	15%	0.15[b]
Within child, between day		0.041	14%		0.060	16%	
Within child, within day		0.185	64%		0.260	69%	
**24-Hour Voids**							
Between child	50	0.028	14%	0.14	0.052	26%	0.26
Within child[c]		0.169	86%		0.148	74%	

[a] Number of samples used in calculation of variance. [b] The ICC presented is the ratio of between-child to total variability as calculated using a one-factor (child) as opposed to a two-factor nested mixed-effects model. [c] Because FMV spots and 24-hour voids allow only one measure per day, the distinction between within-day versus between-day variability is not applicable.
